# Reduced Versus Full‐Dose Direct Oral Anticoagulants for Venous Thromboembolism in Cancer Patients: A Systematic Review and Meta‐Analysis

**DOI:** 10.1002/jha2.70155

**Published:** 2025-09-24

**Authors:** Danyal Bakht, Muhammad Arham, Zarwa Rashid, Maaz Amir, Zarish Nasir, Mustabeen Zahra Naqvi, Maleeha Tahir, Musab Khalil, Esha Gulzar, Hafiz Muhammad Haris, Kinza Bakht, Allah Dad, Haseeb Tareen, Muhammad Numan Awais

**Affiliations:** ^1^ King Edward Medical University Mayo Hospital Lahore Punjab Pakistan; ^2^ Sheikh Zayed Medical College Rahim Yar Khan Punjab Pakistan; ^3^ Henry Ford Health Jackson Michigan USA; ^4^ Shaheed Ziaur Rahman Medical College and Hospital Bogura Bangladesh

## Abstract

**Background:**

Venous thromboembolism (VTE) is a serious complication in cancer patients, with malignancy increasing the risk significantly. Direct oral anticoagulants (DOACs) have emerged as a convenient alternative to traditional therapies, though optimal dosing remains uncertain.

**Methods:**

We performed a systematic review and meta‐analysis on three studies. A comprehensive literature search was performed on PubMed, Embase, the Cochrane Library, and ScienceDirect till April 2025. Analysis was carried out on RevMan 5.4. The risk of bias was assessed via RoB 2.0.

**Results:**

A total of three studies with 2416 participants were identified, including 1495 patients in the reduced‐dose group and 1232 patients in the full‐dose group. No significant difference was observed in recurrent VTE (OR 0.70, 95% CI 0.45–1.09, *p* = 0.11) or recurrent symptomatic VTE (OR 0.96, 95% CI 0.50–1.84, *p* = 0.91). However, reduced‐dose DOACs were associated with a significantly lower incidence of incidental VTE (OR 0.31, 95% CI 0.14–0.69, *p* = 0.004). The reduced‐dose group also had a lower incidence of CRNMB plus major bleeding (OR 0.69, 95% CI 0.55–0.88, *p* = 0.002).

**Conclusions:**

In terms of venous thromboembolism, bleeding events, and all‐cause mortality, reduced‐dose DOACs demonstrated a safety profile that was either superior or comparable to that of full‐dose DOACs.

**Trial Registration:**

The authors have confirmed clinical trial registration is not needed for this submission

**Abbreviations**
API‐CAT: apixaban for cancer‐associated thrombosis
bid: twice daily
BMI: body mass index
CAP: cancer‐associated thrombosis prophylaxis
CRNMB: clinically relevant non‐major bleeding
DOAC: direct oral anticoagulant
DVT: deep vein thrombosis
ECOG: Eastern Cooperative Oncology Group
EVE: extending venous thromboembolism secondary prevention with apixaban in cancer patients
FE: fixed‐effect
GI: gastrointestinal
*I* ^2^
: heterogeneity statistic
ISTH: International Society on Thrombosis and Haemostasis
LMWH: low molecular weight heparin
MD: mean difference
N/A: not applicable
OD: once daily
ONCO‐DVT: edoxaban for cancer‐associated isolated distal deep vein thrombosis
OR: odds ratio
PE: pulmonary embolism
*P*_h_
: *p* value for heterogeneity
PRISMA: Preferred Reporting Items for Systematic Reviews and Meta‐Analyses
PROSPERO: International Prospective Register of Systematic Reviews
RCT: randomized controlled trial
RENOVE: reduced‐dose versus full‐dose direct oral anticoagulants in venous thromboembolism
RevMan: Review Manager
RoB: Risk‐of‐bias
SD: standard deviation
VTE: venous thromboembolism

## 1 Introduction

Venous thromboembolism (VTE), encompassing deep vein thrombosis (DVT) and pulmonary embolism (PE), represents a significant and life‐threatening complication in oncology patients [1]. The incidence of VTE is markedly elevated in individuals with malignancy, with cancer conferring a four‐ to seven‐fold increased risk compared to the general population [2]. This heightened thrombotic risk arises from a complex interplay of factors, including cancer‐induced hypercoagulability, tumor secretion of pro‐coagulant substances (e.g., tissue factor), immobility associated with cancer‐related surgery, and the thrombogenic potential of certain cancer therapies, such as tamoxifen [3].

In recent years, the use of direct oral anticoagulants (DOACs) has emerged as a promising alternative to traditional anticoagulants—namely low molecular weight heparin (LMWH) and warfarin—in the management of cancer‐associated thrombosis (CAT) [4]. DOACs, including rivaroxaban, apixaban, edoxaban, and dabigatran, have gained clinical traction due to their favorable pharmacologic profile [5]; characterized by oral administration, fixed dosing, reduced need for routine monitoring, fewer drug–drug interactions, and a comparatively lower risk of major bleeding events [6].

Despite their growing adoption, the optimal dosing regimen of DOACs in oncology remains a subject of ongoing debate. Current evidence presents a dichotomy between the use of conventional (full) dosing and reduced (off‐label) dosing strategies [7]. Full‐dose regimens are associated with a significantly decreased incidence of recurrent thrombotic events; however, this benefit is offset by an increased risk of major bleeding, particularly in patients with gastrointestinal (GI) malignancies or impaired renal function [8]. Conversely, reduced‐dose DOACs demonstrate a more favorable bleeding profile but may compromise thromboembolic control, raising concerns about efficacy [9].

The lack of robust, conclusive data guiding dosage selection has led to heterogeneous prescribing practices, with some clinicians adopting empiric dose reductions in high‐risk populations despite limited supporting evidence [10]. This emphasizes the critical need for well‐structured research to evaluate the trade‐offs between full and reduced doses of DOACs in cancer patients. A comprehensive evaluation of dosing strategies is imperative to establish an evidence‐based approach that optimally balances thrombotic protection with bleeding risk mitigation. In this systematic review and meta‐analysis, we will evaluate and compare the safety profiles (including bleeding risks) and efficacy outcomes (such as VTE) of full‐dose versus reduced‐dose (DOACs in patients with cancer).

## 2 Materials and Methods

This systematic review and meta‐analysis was conducted in accordance with the Preferred Reporting Items for Systematic Reviews and Meta‐Analyses (PRISMA) 2020 guidelines to ensure methodological rigor and transparency in reporting [11]. Prior to conducting this review, the study protocol was prospectively submitted and registered with International Prospective Register of Systematic Reviews (PROSPERO), with the assigned registration number CRD420251027460.

### 2.1 Search Strategy

A comprehensive and systematic literature search was conducted across four major electronic databases—PubMed, Cochrane Library, ScienceDirect, and Embase—from their inception through April 2025. The search strategy employed a combination of controlled vocabulary and free‐text terms, including the keywords “neoplasm,” “venous thromboembolism,” “DOAC,” “full dose,” “low dose,” and their synonymous or related terms. Boolean operators “AND” and “OR” were utilized to optimize the sensitivity and specificity of the search. In addition, a manual search of the reference lists from previously published meta‐analyses and systematic reviews was performed to identify any potentially eligible studies not captured through the electronic search. The complete search strategy is detailed in Supporting Information S1.

### 2.2 Eligibility Criteria

Studies were eligible for inclusion in this meta‐analysis if they (1) enrolled patients with active malignancy at high risk for VTE; (2) evaluated DOAC therapy; and (3) provided a comparative analysis of conventional/full‐dose versus reduced‐dose DOAC regimens. In addition, studies were required to report at least one predefined clinical outcome of interest.

Studies were excluded if they (1) were case reports, review articles, or involved non‐cancer populations; (2) evaluated non‐DOAC anticoagulants (e.g., heparin and warfarin); (3) compared different treatment durations rather than dosing regimens (full‐ vs. reduced‐dose DOACs); (4) provided insufficient or incomplete outcome data; (5) were published in non‐English languages; or (6) were unavailable as full‐text articles.

### 2.3 Data Extraction

Data extraction was performed independently by two reviewers (M.Am. and M.T.) using a standardized data collection form developed in Google Sheets. Extracted variables included key study characteristics (first author, year of publication), trial name (e.g., apixaban for cancer‐associated thrombosis [API‐CAT], ONCODVT), anticoagulant agent utilized, specific reduced‐dose and full‐dose regimens, sample sizes, specific baseline characteristics, and reported clinical outcomes. Any discrepancies or inconsistencies in data extraction were resolved through discussion and consensus, and, when necessary, by consultation with a third independent reviewer (H.M.H.).

### 2.4 Outcomes

The outcomes assessed in these studies were classified into primary and secondary endpoints. The study's primary outcomes included three VTE‐related endpoints: recurrent VTE (composite endpoint), symptomatic VTE, and incidental/asymptomatic VTE. Recurrent VTE encompassed objectively confirmed symptomatic or incidental DVT/PE, per International Society on Thrombosis and Haemostasis (ISTH) criteria. Symptomatic recurrent VTE required new/worsening symptoms with imaging confirmation, while asymptomatic recurrence involved new/worsening thrombi on imaging (e.g., CT and ultrasonography) without symptoms. The secondary outcomes comprised all additional endpoints not specified as primary. Major bleeding was defined per ISTH criteria as fatal bleeding, symptomatic bleeding in a critical organ, or bleeding causing hemoglobin drop ≥ 2 g/dL or transfusion of ≥ 2 blood units. Clinically relevant non‐major bleeding (CRNMB) included overt bleeding requiring medical intervention, hospitalization, or treatment modification without meeting major bleeding criteria. Clinically relevant bleeding comprised the composite of major bleeding and CRNMB during follow‐up. Fatal bleeding denoted death within 7 days of a major bleed without other causes.

### 2.5 Quality Assessment

The Cochrane risk‐of‐bias tool (RoB 2.0) was used to assess bias risk by evaluating six domains namely: (1) randomization process, (2) intended interventions, (3) missing outcome data, (4) measurement of the outcome, (5) selection of the reported result, and (6) overall risk of bias. Each domain was categorized as “low risk,” “some concerns,” and “high risk” according to the predefined criteria [12].

### 2.6 Statistical Analysis

Meta‐analyses were conducted using Cochrane's Review Manager (RevMan), version 5.4. Pooled direct comparisons were performed between reduced‐dose and full‐dose DOACs. Effect estimates for dichotomous outcomes were calculated as odds ratios (ORs) with corresponding 95% confidence intervals (CIs). Statistical heterogeneity across studies was assessed using the heterogeneity statistic (*I* ^2^), with thresholds interpreted as follows: low heterogeneity (*I* ^2^ < 50%) warranted the use of a fixed‐effects (FEs) model, whereas moderate to high heterogeneity (*I* ^2^ ≥ 50%) necessitated application of a random‐effects model. Prespecified sensitivity analyses were considered to explore potential sources of heterogeneity, including variations in study quality, anticoagulant dosing regimens, and clinical outcomes. A *p* value < 0.05 was considered indicative of statistical significance.

## 3 Results

### 3.1 Systematic Process of Study Selection

**FIGURE 1:**
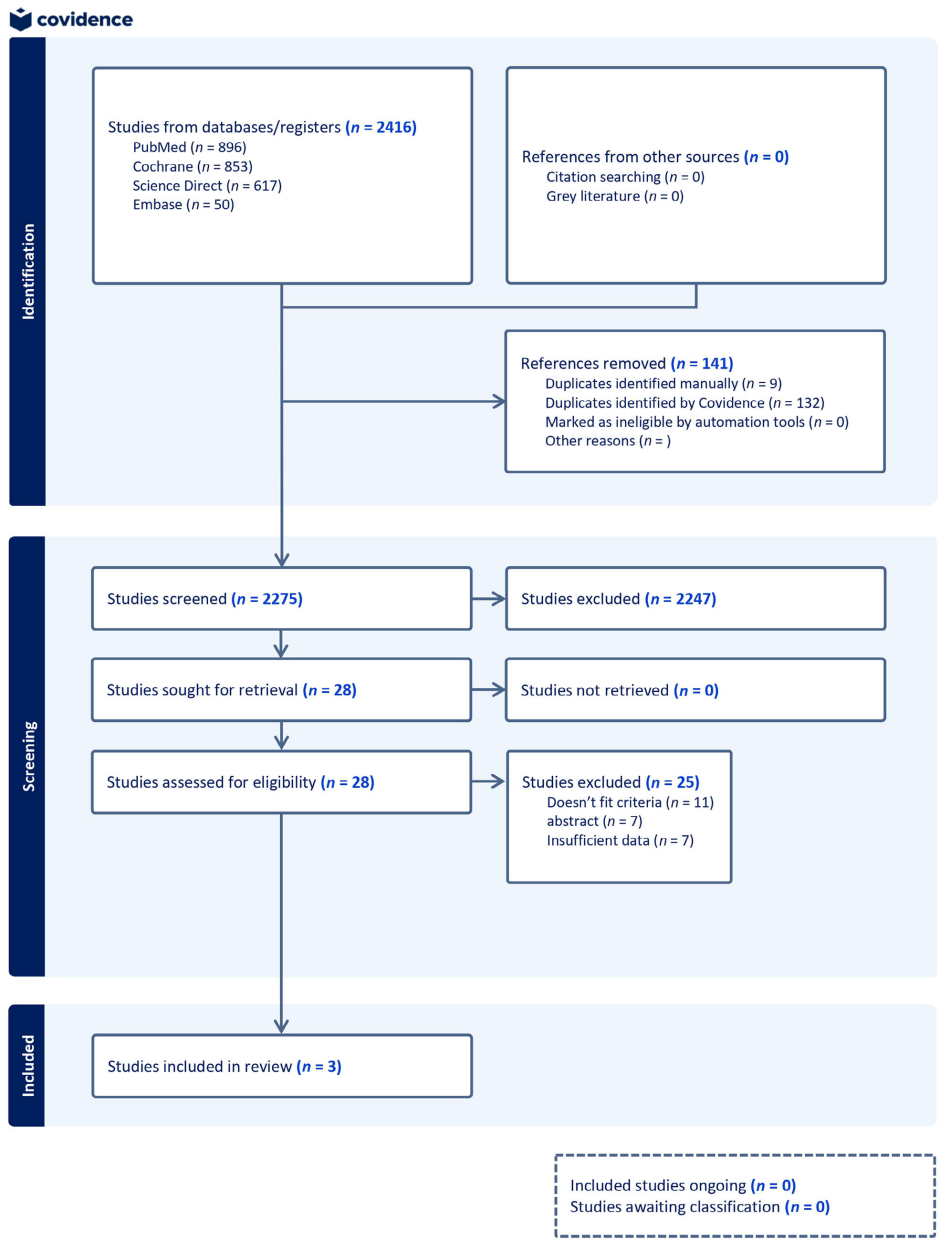
Flow diagram for the systematic process of study selection.

Following the PRISMA guidelines, a total of 2416 records were identified through systematic searches across four databases: PubMed (*n* = 896), Cochrane Library (*n* = 853), ScienceDirect (*n* = 617), and Embase (*n* = 50). After removing 141 duplicate records, 2275 unique studies underwent title and abstract screening. Of these, 2247 were excluded based on predefined eligibility criteria. Twenty‐eight full‐text articles were assessed for eligibility. Twenty‐five studies were subsequently excluded for reasons including nonconformance with the PICO framework (*n* = 11), the availability of abstract only (*n* = 7), and insufficient extractable data (*n* = 7). Ultimately, three randomized controlled trials were included in the qualitative and quantitative synthesis (Figure 1).

### 3.2 Summary of Reviewed Articles

This systematic review and meta‐analysis included three randomized controlled trials (RCTs) involving 2727 patients with neoplasms, requiring long‐term anticoagulation. Of these, 1495 patients (54.9%) were assigned to reduced‐dose DOAC regimens, and 1232 (45.1%) received extended‐ or conventional‐dose regimens. The trials evaluated the efficacy and safety of reduced‐dose versus conventional‐dose DOACs, specifically apixaban (API‐CAT [8] and EVE trials [13]) and edoxaban (edoxaban for cancer‐associated isolated distal deep vein thrombosis [ONCO‐DVT trial] [14]). Dosing regimens were as follows: API‐CAT and EVE trials used apixaban 2.5 (reduced dose) versus 5.0 mg (conventional dose). The ONCO‐DVT trial used edoxaban 30 (reduced dose) versus 60 mg (conventional dose). Baseline study and patient characteristics are summarized in Table 1.

**TABLE 1:** Baseline study and patient characteristics.

	API‐CAT Trial [8]		EVE Trial [13]		Onco‐DVT Trial [14]	

Study characteristics						
Author, year	Mahé et al. 2025		McBane et al. 2024		Chatani et al. 2024	
DOAC	Apixaban		Apixaban		Endoxaban	
Randomization	Reduced dose group (2.5 mg bid)	Extended dose group (5 mg bid)	Reduced dose group (2.5 mg bid)	Extended dose group (5 mg bid)	Reduced dose group (30 mg OD)	Extended dose group (60 mg OD)
Patient characteristics						
Sample size	866	900	179	181	450	151
Age (years), mean ± SD	67.2 ± 11.0	67.7 ± 11.4	63.6 ± 11.0	64.3 ± 10.7	71.9 ± 9.7	67.6 ± 9.8
Gender, male (*n*)	375	391	87	74	86	81
BMI kg/m^2^, mean ± SD	27.0 ± 5.3	27.0 ± 5.4	29.2 ± 6.6	29.5 ± 6.8	21.1 ± 3.0	26.7 ± 4.0
Metastatic disease, *n* (%)	574 (66.3)	584 (64.9)	104 (58.1)	111 (61.3)	113 (25)	34 (23)
History of VTE, *n* (%)	157 (18.1)	170 (18.9)	18 (10.1)	16 (8.8)	23 (5.1)	10 (6.6)
Platelet count < 100,000 per microliter, *n* (%)	18 (2.1)	15 (1.7)	—	—	22 (4.9)	9 (6.0)
Creatinine clearance ≤ 50 mL/min, *n* (%)	115 (13.3)	127 (14.1)	—	—	128 (28)	3 (2.0)
ECOG performance status, *n* (%)						
ECOG performance status, 0	456 (52.7)	504 (56.0)	97 (54.2)	96 (53.0)	223 (50)	88 (58)
ECOG performance status, 1	342 (39.5)	329 (36.6)	73 (40.8)	80 (44.2)	138 (31)	43 (29)
ECOG performance status ≥ 2	67 (7.7)	63 (7.0)	9 (5.0)	5 (2.8)	89 (20)	20 (13)

Abbreviations: BMI, body mass index; bid, twice daily; DOAC, direct oral anticoagulant; ECOG, Eastern Cooperative Oncology Group; OD, once daily; RCT, randomized controlled trial; SD, standard deviation; VTE, venous thromboembolism.

### 3.3 Recurrent Venous Thromboembolism

**FIGURE 2:**
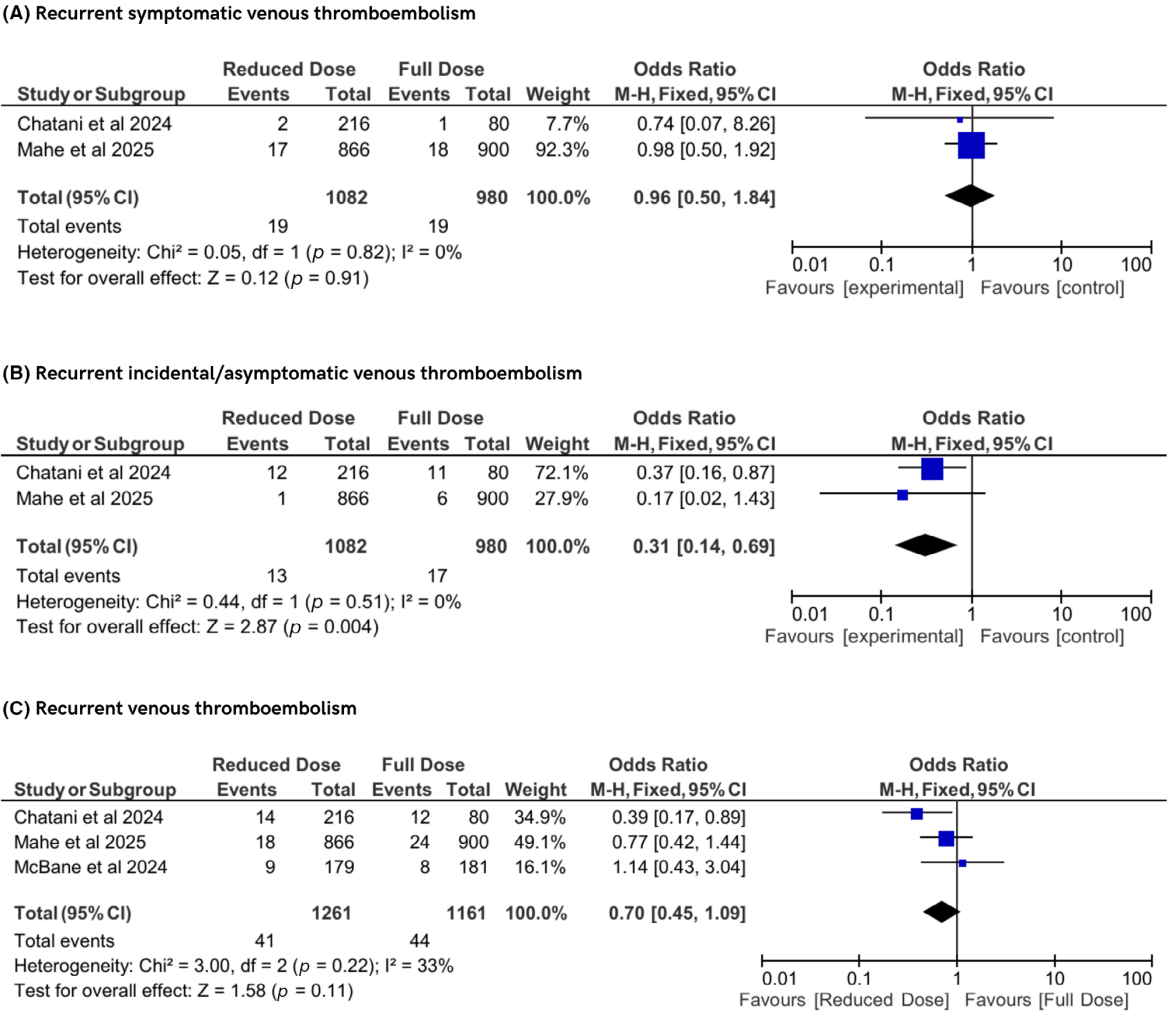
Forest plot of recurrent venous thromboembolism outcomes.

The meta‐analysis assessed key VTE outcomes across the included randomized controlled trials. For recurrent symptomatic VTE, data from two studies showed no significant difference between reduced‐dose and conventional‐dose DOAC regimens (OR 0.96, 95% CI 0.50–1.84, *p* = 0.91), with no heterogeneity (*I* ^2^ = 0%, *P*_h_ = 0.82). Similarly, for recurrent incidental or asymptomatic VTE, two studies indicated a significant reduction in events with reduced‐dose DOACs (OR 0.31, 95% CI 0.14–0.69, *p* = 0.004), with no heterogeneity (*I* ^2^ = 0%, *P*_h_ = 0.51). For the composite outcome of recurrent VTE, encompassing three studies, no significant difference was observed between dosing regimens (OR 0.70, 95% CI 0.45–1.09, *p* = 0.11), with moderate heterogeneity (*I* ^2^ = 33%, *P*_h_ = 0.22). These findings suggest that reduced‐dose DOACs may reduce incidental VTE events while maintaining comparable efficacy to conventional doses for symptomatic and overall VTE recurrence (Figure 2).

### 3.4 VTE Sub‐Types

**FIGURE 3:**
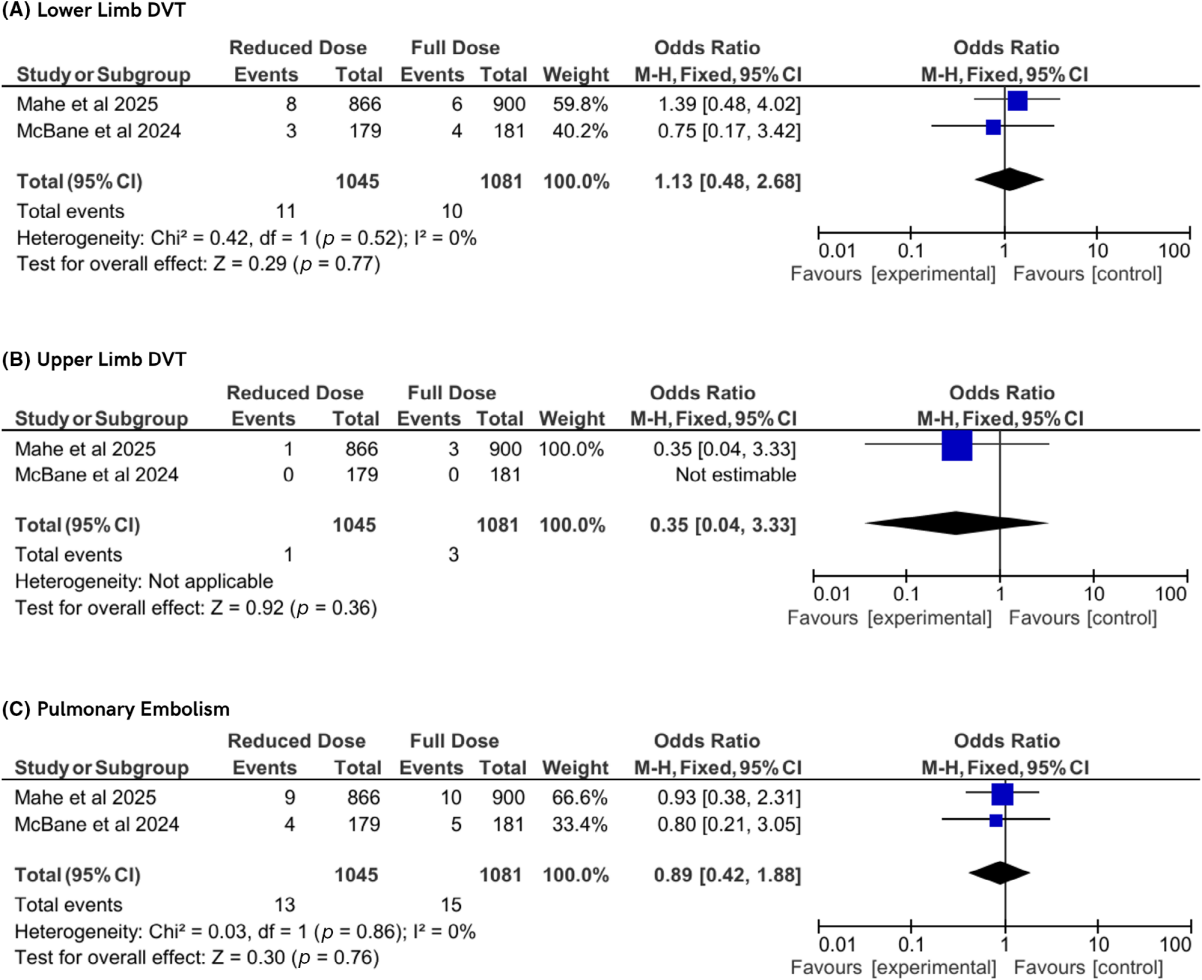
Forest plot of VTE subtypes.

This meta‐analysis also evaluated secondary outcomes related to specific venous VTE subtypes, bleeding events, and mortality across the included randomized controlled trials. For lower limb DVT, data from two studies showed no significant difference between reduced‐dose and conventional‐dose DOAC regimens (OR 1.13, 95% CI 0.48–2.68, *p* = 0.77), with no heterogeneity (*I* ^2^ = 0%, *P*_h_ = 0.52). Similarly, upper limb DVT, assessed in two studies, showed no significant difference (OR 0.35, 95% CI 0.04–3.33, *p *= 0.36), with heterogeneity not applicable due to limited events. PE, evaluated in two studies, also showed no significant difference (OR 0.89, 95% CI 0.42–1.88, *p* = 0.76), with no heterogeneity (*I* ^2^ = 0%, *P*_h_ = 0.86). These findings suggest reduced‐dose DOACs demonstrate similar efficacy to conventional dosing for preventing certain VTE subtypes (Figure 3).

### 3.5 Bleeding Outcomes

**FIGURE 4:**
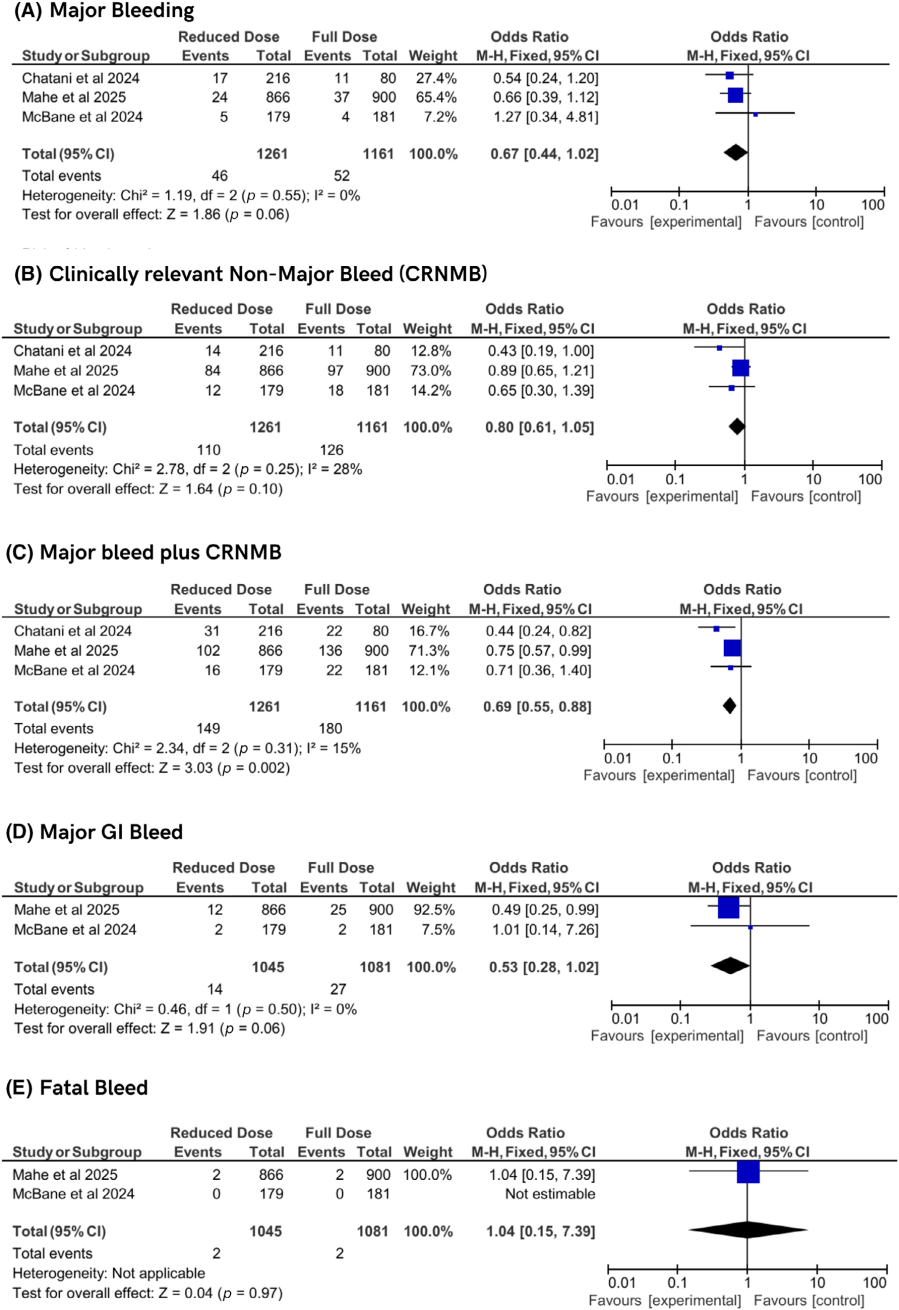
Forest plot of bleeding outcomes.

Bleeding outcomes were analyzed across multiple endpoints. Major bleeding, assessed in three studies, showed no significant difference between the two groups (OR 0.67, 95% CI 0.44–1.02, *p* = 0.06), with no heterogeneity (*I* ^2^ = 0%, *P*_h_ = 0.55). CRNMB, evaluated in three studies, showed no significant difference (OR 0.80, 95% CI 0.61–1.05, *p* = 0.10), with moderate heterogeneity (*I* ^2^ = 28%, *P*_h_ = 0.25). The composite outcome of major bleeding plus CRNMB, also from three studies, demonstrated a significant reduction with reduced‐dose regimens (OR 0.69, 95% CI 0.55–0.88, *p* = 0.002), with low heterogeneity (*I* ^2^ = 15%, *P*_h_ = 0.31). Major GI bleeding, assessed in two studies, showed a nonsignificant reduction with reduced‐dose DOACs (OR 0.53, 95% CI 0.28–1.03, *p* = 0.06), with no heterogeneity (*I* ^2^ = 0%, *P*_h_ = 0.50). Fatal bleeding, evaluated in two studies, showed no difference (OR 1.04, 95% CI 0.15–7.37, *p* = 0.97), with heterogeneity not applicable due to rare events. These findings suggest a potential safety advantage for reduced‐dose DOACs, particularly for the composite bleeding outcome (Figure 4).

### 3.6 All‐Cause Mortality

**FIGURE 5:**
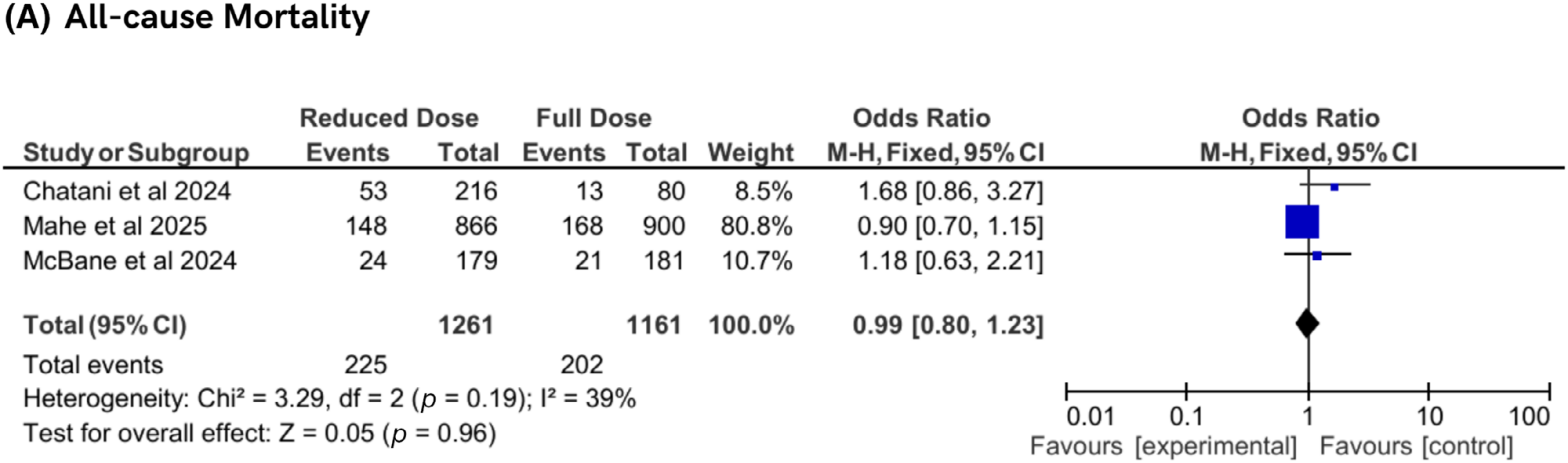
Forest plot of all‐cause mortality.

All‐cause mortality was assessed in three studies and showed no significant difference between reduced‐dose and conventional‐dose DOAC regimens (OR 0.99, 95% CI 0.80–1.23, *p* = 0.96), with moderate heterogeneity (*I* ^2^ = 39%, *P*_h_ = 0.19). This indicates that reduced‐dose DOACs do not impact overall mortality compared to conventional dosing, maintaining a neutral effect on this outcome (Figure 5). Table 2 displays a comprehensive overview of all primary and secondary outcomes with their associated results.

**TABLE 2:** Meta‐analytic outcomes of recurrent venous thromboembolism, VTE subtypes, bleeding events, and all‐cause mortality.

Outcomes	No. of Studies	Effect model	MD/OR	*I* ^2^ (%)	*P*_h_	Effect size	*p* value
Recurrent venous thromboembolism (VTE)							
Recurrent symptomatic VTE	2	FE	OR	0	0.82	0.96 (0.50, 1.84)	0.91
Recurrent incidental/asymptomatic VTE	2	FE	OR	0	0.51	0.31 (0.14, 0.69)	0.004
Recurrent VTE	3	FE	OR	33%	0.22	0.70 (0.45, 1.09)	0.11
VTE‐subtype							
Lower limb DVT	2	FE	OR	0	0.52	1.13 (0.48, 2.68)	0.77
Upper limb DVT	2	FE	OR	N/A	N/A	0.35 (0.04, 3.33)	0.36
Pulmonary embolism	2	FE	OR	0	0.86	0.89 (0.42, 1.88)	0.76
Bleeding outcomes							
Major bleeding	3	FE	OR	0	0.55	0.67 (0.44, 1.02)	0.06
Clinically relevant non‐major bleed (CRNMB)	3	FE	OR	28%	0.25	0.80 (0.61, 1.05)	0.10
Major bleed plus CRNMB	3	FE	OR	15%	0.31	0.69 (0.55, 0.88)	0.002
Major GI bleed	2	FE	OR	0	0.50	0.53 (0.28, 1.03)	0.06
Fatal bleeding	2	FE	OR	N/A	N/A	1.04 (0.15, 7.37)	0.97
Mortality outcomes							
Death from any cause	3	FE	OR	39%	0.19	0.99 (0.80, 1.23)	0.96

Abbreviations: CRNMB, clinically relevant non‐major bleeding; DVT, deep vein thrombosis; FE, fixed effect; GI, gastrointestinal; *I* ^2^, heterogeneity statistic; MD, mean difference; OR, odds ratio; *P*_h_, *p* value for heterogeneity; VTE, venous thromboembolism.

### 3.7 Risk of Bias Assessment

**FIGURE 6:**
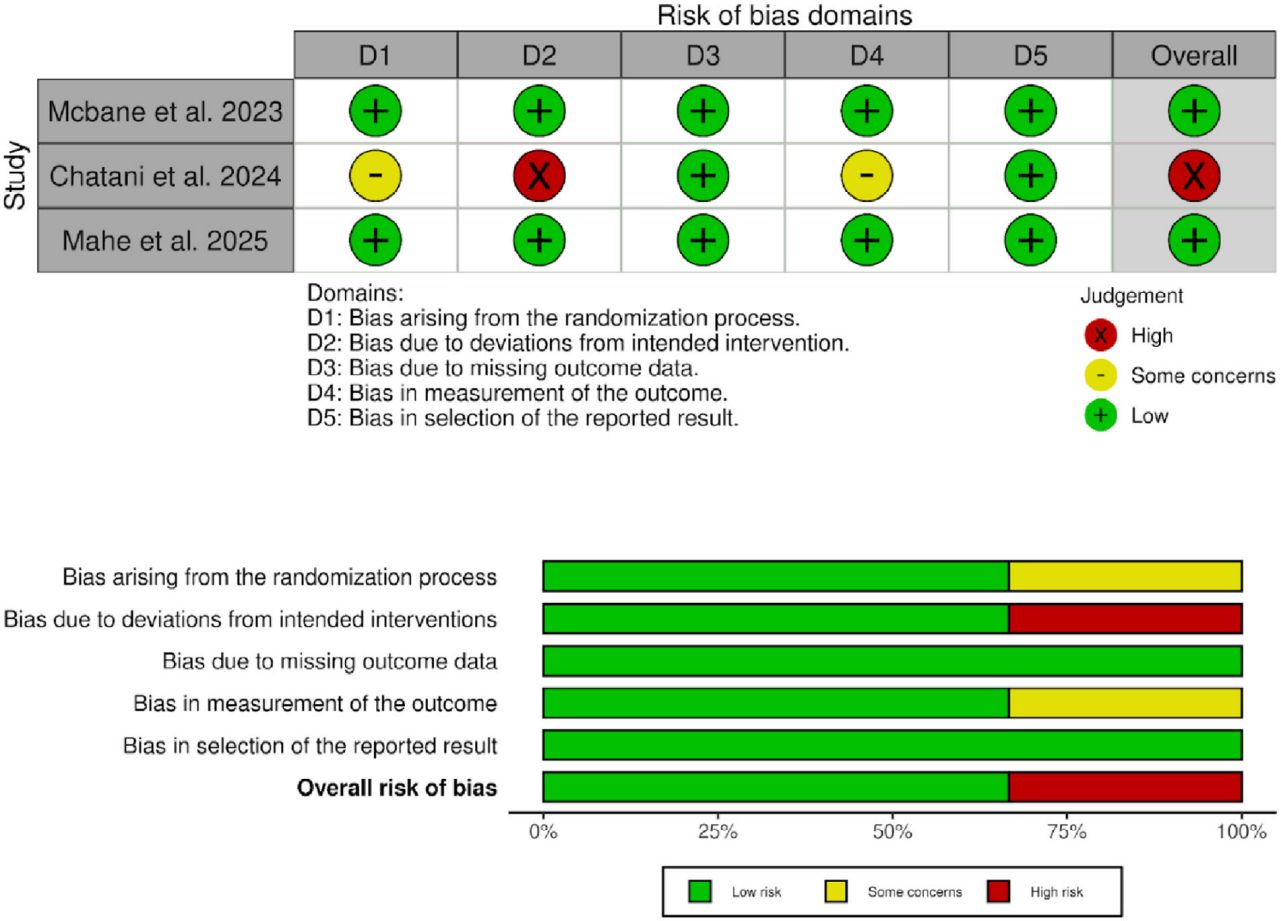
The risk of bias assessment ‐ using RoB‐2.0. The traffic plot and summary plot were drawn using robvis (visualization tool).

We evaluated study quality using the Cochrane Risk of Bias tool (RoB 2.0). Among the included studies, two demonstrated low risk of bias, while one study was rated as having high risk (Figure 6).

### 3.8 Sensitivity Analysis

Given the absence of substantial heterogeneity (*I* ^2^ < 50%) across the included studies, sensitivity analysis was deemed methodologically unwarranted.

### 3.9 Publication Bias

In accordance with recommended methodological guidelines, formal evaluation of publication bias using Egger's test was not performed, as such statistical approaches are considered unreliable in meta‐analyses with fewer than 10 included studies [15].

## 4 Discussions

This systematic review and meta‐analysis, incorporating data from three RCTs with 2416 patients, evaluated the comparative safety and efficacy of reduced‐dose versus conventional‐dose DOACs in patients with malignancy‐associated VTE. The findings demonstrated that reduced‐dose DOAC regimens offered similar protection against recurrent symptomatic VTE events compared to conventional doses, with no statistically significant difference. Interestingly, reduced doses were associated with a significant reduction in incidental or asymptomatic VTE, and a favorable trend was observed in composite bleeding outcomes, including a statistically significant reduction in combined major and CRNMB events. No difference was observed in all‐cause mortality or VTE subtypes such as DVT or PE. These findings suggest that reduced‐dose DOACs may provide a clinically viable and potentially safer alternative for long‐term anticoagulation in cancer patients at risk for thromboembolic events.

The findings from the three trials, included in our meta‐analysis show some variability in outcomes. The EVE trial by McBane et al. [13] and API‐CAT trial by Mahé et al. [8] found no significant difference in recurrent VTE rates between reduced‐dose and full‐dose apixaban. In contrast, the ONCO DVT study by Chatani et al. [14] demonstrated a significant reduction in recurrent VTE with a 12‐month treatment regimen, irrespective of the edoxaban dose, while also showing a marked increase in major bleeding with the full‐dose regimen, suggesting that the duration of treatment plays a crucial role. Mortality rates remained similar between reduced and full‐dose groups in both the EVE and API‐CAT trials. The variability in results can be attributed to differences in treatment duration, anticoagulant agents used, and patient populations.

The findings of a recent meta‐analysis by Vasanthamohan et al. [10], that assesses the effects of reduced dose DOACs in patients with VTE not specifically cancer patients, broadly align with our results in terms of recurrent VTE, as both analyses showed that reduced‐dose DOACs offer similar efficacy to full‐dose regimens for preventing VTE recurrence. Vasanthamohan et al. [10] observed a trend toward reduced bleeding with reduced‐dose DOACs, but this did not reach statistical significance. In contrast, our meta‐analysis showed a clear and significant reduction in the composite of major and CRNMB events. This divergence may be attributed to the higher baseline bleeding risk in oncology patients, where even modest dose reductions can translate into meaningful clinical benefits. As for all‐cause mortality, both meta‐analyses reported no significant difference between reduced‐ and full‐dose strategies.

While it may seem that reduced‐dose and full‐dose oral anticoagulants might be comparable in terms their efficacy as indicated by the findings of our meta‐analysis and the one by Vasanthamohan et al. [10], literature also reveals contradictory evidence. The reduced‐dose versus full‐dose direct oral anticoagulants in VTE (RENOVE) trial, a randomized multicenter study, evaluated reduced‐dose versus full‐dose DOACs in patients requiring extended anticoagulation for VTE after 6–24 months of standard treatment. While the reduced‐dose regimen did not statistically meet the non‐inferiority criteria for preventing recurrent VTE, both arms showed very low recurrence rates. These findings support the consideration of dose‐reduced DOACs as a viable strategy for long‐term anticoagulation in selected patients, particularly those at high‐bleeding risk [9].

Our meta‐analysis was designed to evaluate the safety and efficacy of reduced‐dose versus full‐dose DOACs specifically within the cancer population, and therefore RENOVE trial that did not focus exclusively on patients with cancer‐associated VTE, was excluded from our study. A significant proportion of patients with VTE face a persistent risk of recurrence, often necessitating extended anticoagulant therapy beyond the initial 3–6 months. However, the decision to prolong treatment must be individualized, weighing the risk of recurrent thromboembolism against the potential for serious bleeding complications [16]. Given the delicate balance between thrombotic risk and bleeding complications in cancer‐associated VTE, strategies to optimize long‐term anticoagulation are of growing interest. The cancer‐associated thrombosis prophylaxis (CAP) study explored this by transitioning cancer patients to low‐dose apixaban after 6 months of full‐dose therapy, demonstrating sustained efficacy with a low risk of bleeding over a 30‐month period indicating that dose reduction may be a viable and safe option for long‐term management in select individuals [17].

In interpreting our findings, it's important to clarify the clinical implications of various reduced‐dose anticoagulation strategies. Reduced‐dose DOAC use can take the form of either dose adjustment, based on patient‐specific criteria like renal function or age, or intentional low‐intensity regimens for VTE prevention. While dose adjustment is generally unsuitable for acute VTE treatment, low‐intensity DOACs may be appropriate for extended prophylaxis in cancer patients [18]. Although reduced‐dose DOACs have shown promise in select patients with cancer‐associated VTE, their use should not be generalized across all clinical scenarios. A recent meta‐analysis investigating off‐label underdosing of DOACs in atrial fibrillation patients revealed significantly increased risks of all‐cause mortality and adverse cardiovascular outcomes compared to standard dosing, albeit based on low‐quality cumulative evidence [19].

It is also important to highlight that variability in the safety and efficacy profiles among individual DOAC agents necessitates agent‐specific evaluation rather than generalizing reduced‐dose outcomes across the entire class. A large registry‐based meta‐analysis in patients with non‐valvular atrial fibrillation demonstrated that apixaban and dabigatran were associated with particularly favorable bleeding outcomes. In contrast, rivaroxaban was linked to an increased risk of major bleeding despite a reduction in stroke incidence, and apixaban showed a slight increase in all‐cause mortality [20]. Although this study was not conducted in the context of cancer‐associated VTE, it highlights the heterogeneity in efficacy and safety across different DOAC agents, thereby reinforcing the need for further research to evaluate agent‐specific outcomes in CAT.

This meta‐analysis suggest that reduced‐dose DOACs can offer a safer, more effective long‐term anticoagulation option for cancer patients with VTE. There is an increase in major bleeding risk with DOAC, particularly observed in GI and potentially genitourinary malignancies [21]. By reducing the dose, clinicians might be able to balance VTE prevention with minimizing bleeding risks, particularly in patients with GI or genitourinary malignancies. This approach may also enhance patient adherence by simplifying treatment regimens [22]. Healthcare providers should consider individualized, risk‐adapted anticoagulation strategies, factoring in cancer type, bleeding risk, and potential drug interactions. Ultimately, this could lead to better patient outcomes and reduced healthcare costs by lowering bleeding‐related complications and hospitalizations.

Several methodological constraints warrant consideration in interpreting these findings. Primarily, the limited number of eligible trials (*n* = 3) inherently restricts the statistical power of our meta‐analysis. Notably, the ONCO‐DVT trial's evaluation of edoxaban introduces potential pharmacological heterogeneity when compared to the apixaban‐based protocols employed in the remaining studies [14]. This inter‐agent variability is further compounded by divergent dosing regimens across studies. The included trials employed incompatible dosing schemes: edoxaban (60→30 mg daily) in ONCO‐DVT versus apixaban (5→2.5 mg twice daily [bid]) in others, creating inherent comparability challenges. In addition, clinical heterogeneity stemming from variations in both malignancy subtypes and therapeutic durations may constrain the external validity of our pooled results.

Future research should aim to validate these findings in larger, multicenter RCTs that stratify patients based on cancer type, stage, and bleeding risk. Trials with longer follow‐up durations and patient‐level data are needed to assess late VTE recurrences and delayed bleeding complications. Further investigation into pharmacogenomics and biomarker‐guided anticoagulation strategies could also refine dosing decisions for DOACs in oncology patients.

## 5 Conclusions

Reduced‐dose DOAC regimens appear to maintain clinical efficiency in preventing recurrent symptomatic VTE while significantly reducing the risk of incidental VTE and composite bleeding events in cancer patients. The absence of differences in all‐cause mortality and VTE subtypes further supports the viability of dose reduction as a long‐term anticoagulation strategy. These findings highlight a potentially safer therapeutic approach for cancer patients at increased bleeding risk. However, limited sample size, variations in trial design, treatment duration, and patient characteristics emphasize the need for further agent‐specific clinical trials to better inform individualized anticoagulation strategies in cancer patients.

## Conflicts of Interest

The authors declare no conflicts of interest.

## Supporting information



Supporting file: jha270155‐sup‐0001‐SuppMat.docx

## Data Availability

No new data were generated for this study. All data used in this meta‐analysis were extracted from previously published studies, which are publicly available and cited accordingly. Further details can be obtained from the corresponding author upon reasonable request.
