# Supplementary material for: Reduced Versus Full‐Dose Direct Oral Anticoagulants for Venous Thromboembolism in Cancer Patients: A Systematic Review and Meta‐Analysis
Source: EJHaem. 2025 Sep 24;6(5):e70155. doi: 10.1002/jha2.70155 (PMC12459301; doi:10.1002/jha2.70155)
Supplement: Supplementary file 1 — Supporting file: jha270155‐sup‐0001‐SuppMat.docx [file JHA2-6-e70155-s001.docx]

**Reduced versus Full-Dose Direct Oral Anticoagulants for Venous Thromboembolism in Cancer Patients: A Systematic Review and Meta-Analysis**

**Authors:**

1. **Danyal Bakht:** MBBS, King Edward Medical University, Mayo Hospital, Lahore, Punjab, Pakistan [danyalbakht190@gmail.com](mailto:danyalbakht190@gmail.com)
2. **Muhammad Arham:** MBBS, Sheikh Zayed Medical College, Rahim Yar Khan, Punjab, Pakistan [z18.mbbs.429@gmail.com](mailto:z18.mbbs.429@gmail.com)
3. **Zarwa Rashid:** MBBS, MD, King Edward Medical University, Mayo hospital, Lahore, Punjab, Pakistan [rashidzarwa@gmail.com](mailto:rashidzarwa@gmail.com)
4. **Maaz Amir:** MBBS, King Edward Medical University, Mayo Hospital, Lahore, Punjab, Pakistan [amirmaaz067@gmail.com](mailto:amirmaaz067@gmail.com)
5. **Zarish Nasir:** MBBS, King Edward Medical University, Mayo Hospital, Lahore, Punjab, Pakistan [nasirzarish7@gmail.com](mailto:nasirzarish7@gmail.com)
6. **Mustabeen Zahra Naqvi:** MBBS, MD, King Edward Medical University, Mayo Hospital, Lahore, Punjab, Pakistan [mustabeen.naqvi@gmail.com](mailto:mustabeen.naqvi@gmail.com)
7. **Maleeha Tahir:** MBBS, King Edward Medical University, Mayo Hospital, Lahore, Punjab, Pakistan [maleeha.tahir011@gmail.com](mailto:maleeha.tahir011@gmail.com)
8. **Musab Khalil:** MBBS, King Edward Medical University, Mayo Hospital, Lahore, Punjab, Pakistan [musab.khalil99@gmail.com](mailto:musab.khalil99@gmail.com)
9. **Esha Gulzar:** MBBS, King Edward Medical University, Mayo Hospital, Lahore, Punjab, Pakistan [eshagulzar3@gmail.com](mailto:eshagulzar3@gmail.com)
10. **Hafiz Muhammad Haris:** MBBS, King Edward Medical University, Mayo Hospital, Lahore, Punjab, Pakistan [hafizmharis@kemu.edu.pk](mailto:hafizmharis@kemu.edu.pk)
11. **Kinza Bakht:** MBBS, Sheikh Zayed Medical College, Rahim Yar Khan, Punjab, Pakistan

[Kinzabakht02@gmail.com](mailto:Kinzabakht02@gmail.com)

1. **Allah Dad:** MBBS, MD, Shiekh Zayed Medical College Rahim Yar Khan, Pakistan [4mbbs928@gmail.com](mailto:4mbbs928@gmail.com)
2. **Haseeb Tareen:** MBBS, MD, Henry Ford Health, Jackson, MI [htareen1@hfhs.org](mailto:htareen1@hfhs.org)
3. **Muhammad Numan Awais (Corresponding Author):** Shaheed Ziaur Rahman Medical college and Hospital, Bogura muhammadnumanawais433@gmail.com

**Corresponding Author: Muhammad Numan Awais:** Shaheed Ziaur Rahman Medical college and Hospital, Bogura. muhammadnumanawais433@gmail.com. Contact: +8801323885761

**Search Strategy**

**PubMed**

("Venous thromboembolism"[MeSh] OR "VTE" OR "deep vein thrombosis" OR "pulmonary embolism" OR "thromboembolism"[MeSh] OR "thromboembolic disease" OR "thromboembolic disorder" OR "thrombosis" OR "embolism") AND ("anticoagulants"[MeSh] OR "Direct Oral Anticoagulants" OR DOAC OR "NOAC" OR "Apixaban" OR "Rivaroxaban" OR "Edoxaban" OR "Dabigatran" OR "factor Xa inhibitors" OR "thrombin inhibitors") AND ("Neoplasms"[MeSh] OR "cancer" OR "malignancy" OR "neoplastic disease" OR "cancer-associated thrombosis" OR "oncologic patients" OR "cancer patients" OR "malignancy-associated thrombosis") AND ("Drug Administration Schedule"[MeSh] OR "dose" OR "low dose" OR "reduced dose" OR "full dose" OR "extended dosing" OR "long-term anticoagulation" OR "extended phase anticoagulation")

**Cochrane Library:**

1. MeSH descriptor: [Venous Thromboembolism] explode all trees
2. "VTE" OR "deep vein thrombosis" OR "DVT" OR "pulmonary embolism" OR "PE"
3. MeSH descriptor: [Thromboembolism] explode all trees
4. "thromboembolic disease" OR "thromboembolic disorder" OR "thrombosis" OR "embolism" OR "venous embolism" OR "arterial thromboembolism"
5. MeSH descriptor: [Anticoagulants] explode all trees
6. "oral anticoagulants" OR "direct oral anticoagulants" OR "DOAC" OR "NOAC" OR "factor Xa inhibitors" OR "thrombin inhibitors" OR "Apixaban" OR "Rivaroxaban" OR "Edoxaban" OR "Dabigatran"
7. MeSH descriptor: [Drug Administration Schedule] explode all trees
8. "extended full dose" OR "standard dose" OR "full-dose anticoagulation" OR "low dose" OR "reduced dose" OR "dose reduction" OR "extended dosing" OR "long-term anticoagulation" OR "maintenance therapy" OR "extended phase anticoagulation"
9. #1 OR #2 OR #3 OR #4
10. #5 OR #6
11. #7 OR #8
12. #9 AND #10 AND #11 AND #12

**Science Direct**

("thromboembolism") AND ("direct oral anticoagulant" OR Apixaban OR Rivaroxaban) AND (cancer OR neoplasm OR malignancy) AND ("low dose" OR "full dose")

**Embase**

('thromboembolism'/exp OR 'cerebral embolism and thrombosis' OR 'embolism and thrombosis' OR 'embolism, thrombo' OR 'intracranial embolism and thrombosis' OR 'thrombo embolic disease' OR 'thrombo embolism' OR 'thrombo-emboli' OR 'thrombo-embolus' OR 'thromboemboli' OR 'thromboembolic' OR 'thromboembolic complication' OR 'thromboembolic disease' OR 'thromboembolic process' OR 'thromboembolism' OR 'thromboembolus' OR 'thromboemboly') AND 'direct oral anticoagulant'/exp)
